# Genotoxicity Assessment of Metal-Based Nanocomposites Applied in Drug Delivery

**DOI:** 10.3390/ma14216551

**Published:** 2021-11-01

**Authors:** Sara Cardoso, Classius F. da Silva, Patrícia Severino, Amélia M. Silva, Selma B. Souto, Aleksandra Zielińska, Jacek Karczewski, Eliana B. Souto

**Affiliations:** 1 Polo das Ciências da Saúde, Departament of Pharmaceutical Technology, Faculty of Pharmacy, University of Coimbra, Azinhaga de Santa Comba, 3000-548 Coimbra, Portugal; SaraCardoso_1996@hotmail.com (S.C.); aleksandra.zielinska@igcz.poznan.pl (A.Z.); 2 Instituto de Ciências Ambientais, Químicas e Farmacêuticas, Federal University of São Paulo, Rua São Nicolau, 210, Diadema 09913-030, Brazil; cfsilva@unifesp.br; 3 Instituto de Tecnologia e Pesquisa, Universidade Tiradentes, Av. Murilo Dantas, 300, Aracaju 49032-490, Brazil; patricia_severino@itp.org.br; 4 Post-Graduation Program in Industrial Biotechnology, Universidade Tiradentes, Av. Murilo Dantas, 300, Aracaju 49032-490, Brazil; 5 Centre for Research and Technology of Agro-Environmental and Biological Sciences (CITAB), University of Trás-os Montes e Alto Douro (UTAD), Quinta de Prados, 5001-801 Vila Real, Portugal; amsilva@utad.pt; 6 Department of Biology and Environment, University of Trás-os-Montes e Alto Douro (UTAD), Quinta de Prados, 5001-801 Vila Real, Portugal; 7 Department of Endocrinology, Hospital de São João, Alameda Prof. Hernâni Monteiro, 4200-319 Porto, Portugal; sbsouto.md@gmail.com; 8 Institute of Human Genetics, Polish Academy of Sciences, Strzeszyńska 32, 60-479 Poznan, Poland; 9 Department of Environmental Medicine, Poznan University of Medical Sciences, 60-355 Poznan, Poland; 10 Department of Gastroenterology, Dietetics and Internal Diseases, H. Swiecicki University Hospital, Poznan University of Medical Sciences, 60-355 Poznan, Poland; 11 CEB—Centro de Engenharia Biológica, Campus de Gualtar, Universidade do Minho, 4710-057 Braga, Portugal

**Keywords:** metal nanoparticles, drug delivery, genotoxicity, comet assay, nanotoxicology, DNA damage

## Abstract

Nanocomposites as drug delivery systems (e.g., metal nanoparticles) are being exploited for several applications in the biomedical field, from therapeutics to diagnostics. Green nanocomposites stand for nanoparticles of biocompatible, biodegradable and non-toxic profiles. When using metal nanoparticles for drug delivery, the question of how hazardous these “virus-sized particles” can be is posed, due to their nanometer size range with enhanced reactivity compared to their respective bulk counterparts. These structures exhibit a high risk of being internalized by cells and interacting with the genetic material, with the possibility of inducing DNA damage. The Comet Assay, or Single-Cell Gel Electrophoresis (SCGE), stands out for its capacity to detect DNA strand breaks in eukaryotic cells. It has huge potential in the genotoxicity assessment of nanoparticles and respective cells’ interactions. In this review, the Comet assay is described, discussing several examples of its application in the genotoxicity evaluation of nanoparticles commonly administered in a set of routes (oral, skin, inhaled, ocular and parenteral administration). In the nanoparticles boom era, where guidelines for their evaluation are still very limited, it is urgent to ensure their safety, alongside their quality and efficacy. Comet assay or SCGE can be considered an essential tool and a reliable source to achieve a better nanotoxicology assessment of metal nanoparticles used in drug delivery.

## 1. Introduction

Nanocomposites as nanoparticulate delivery systems are playing a major role in the development of new drug formulations, as they can deliver a substance to the target with higher efficiency and precision than conventional forms, avoiding possible undesirable effects [1]. Other attributes, such as the potential use as theragnostic agents, have also been described [1,2]. However, a new concern has been raised as a consequence of the growing production and application of nanoparticles in drug delivery. “Nanotoxicology”, how dangerous nanoparticles can be? “Small” also means that nanoparticles can reach places that other larger particles cannot, such as the cellular core, where DNA is, which implies that they may interact with genetic material [3]. While the use of nanoparticles can be applied for DNA damage to remove neoplastic cells and cause cell death, miss-repaired damage or the occurrence of other nanoparticle interactions with the genetic material can alter the cells’ functions and interfere with the synthesis of proteins, which may cause potential diseases or even lead to carcinogenicity [4].

Genetic material-related toxicity is known as “genotoxicity” and the potential of nanoparticles to induce genotoxicity can be considered a primary (direct or indirect) or a secondary interaction. The genotoxicity effect can be direct if the nanoparticles exhibit the capacity to reach the nucleus and cause lesions directly in the genetic material. Indirect damage happens due to their capacity to induce oxidative stress which can cause genotoxicity. Secondary DNA damage may occur, for example, due to the capacity of macrophages and/or neutrophils to cause an inflammatory response, which is based essentially on the release of inflammatory cytokines, causing cellular lesions that can be reflected in the integrity of genetic material [5].

Despite the increasing research focusing on this topic, studies are still very limited and possible hazardous effects associated with nanoparticles are still unknown [6]. Therefore, efforts are currently being made to further assess their safety, especially because these “virus-sized particles” are in continuous contact with humans daily [7].

While official guidelines for evaluating the safety of nanoparticles are still somewhat limited, regulatory authorities have been making an effort to implement recommendations on nanotoxicology assessment [8]. Genotoxicity tests are an extremely important portion of this assessment. Among the available tests, the Comet Assay is currently in use both in vitro and in vivo to measure genotoxicity. It is already considered a powerful and promising tool for assessing DNA damage in clinical research and a standard application in the pharmaceutical industry for the evaluation of the safety profile of new drug formulations [9,10,11]. This assay was not specifically designed for nanoparticles [12], but its potential to explore their genotoxicity assessment is mentioned by several authors. Numerous organizations recommend the comet assay. In 2014, the OECD (Organization for Economic Co-Operation and Development) created the 489 guideline for the “In vivo Mammalian Alkaline Comet Assay”, which describes the method in detail, as well its limitations and considerations, historical control data, and other information to consider [13]. The International Conference on Harmonization of Technical Requirements for Registration of Pharmaceuticals for Human Use (ICH) also recommends its practice, among other assays, for a broader in vivo assessment [14]. Additionally, the European Food Safety Authority (EFSA) recognizes the importance of this assay, and it is recommended as a suitable approach by the Registration, Evaluation Authorization, and Restriction of Chemicals program of the European Commission [12,14].

Although an in vitro comet assay is not yet included in regulatory assessment toxicity guidelines, investments are being made to validate its use. Attributed to its versatility, robustness and reliability, it is likely to be included in a test battery for genotoxicity assessment [12].

## 2. The Comet Assay

The single-cell gel electrophoresis (SCGE), commonly known as comet assay, measures DNA strand breaks, at the level of individual eukaryotic cells [15]. It is a simple and sensitive method, frequently performed in animal cells, whether in culture or isolated from the organism, however, examination of DNA damage in plant cells is also possible [16]. This procedure, developed by Östling and Johanson (1984) and then adapted and optimized by Singh et al., (1988), is being considered one of the standard methods, not only for assessing DNA damage, with applications in human biomonitoring, genotoxicity testing, molecular epidemiology and ecotoxicology [15,17], but also to evaluate the DNA repair ability of cells [12], since the incubation of a damaging agent with cells can be monitored by measuring the damage remaining at intervals [15].

Depending on the literature and according to the purpose of this procedure, there are several ways of performing it, but the method most commonly used is the alkaline comet assay (Singh et al. procedure), for being the most sensitive in terms of detecting strand breaks of DNA, compared to the neutral one (Östling and Johanson procedure) [9]. Essentially, the alkaline process (pH > 13) detects not only single-strand breaks (SSBs) and double-strand breaks (DSBs) and even alkali labile sites (ALSs), but also, through the combination of specific endonucleases, particular base lesions [9]. Neutral SCGE only detects DSBs.

The alkaline comet assay is a very sensitive method, as it detects low levels of DNA damage [18], however, several other advantages such as the requirement for small numbers of cells per sample, low cost, flexibility, performance facility and short assay execution time characterize this procedure [19]. Additionally, this assay considers both DNA content and DNA damage, allowing the measurement of the damage at any phase of the cell cycle [13].

**Figure 1:**
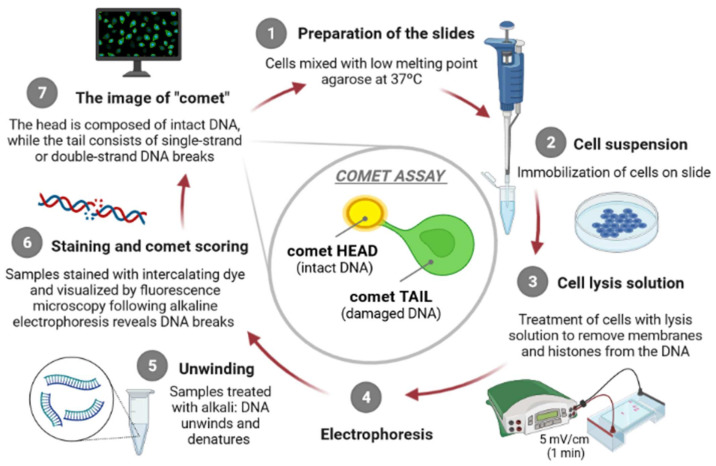
The principle of the comet assay [own drawing]. The alkaline comet assay is mainly consisted of seven steps, including preparation of the microscope slides (1), suspension of cells (2), lysis of cells (3), exposure to alkali (pH > 13) and electrophoresis (4), neutralization of alkali (5), DNA staining (6) and finally, comet visualization and scoring (7).

The principle of the comet assay, shown in Figure 1, consists in incorporating cells in agarose gel layers on microscope slides, placing them in the presence of high salts concentration and detergents to occur the lysis of the cells, generating “nucleoids”. At this point, DNA organization consists of negatively supercoiled loops anchored to a residual proteinaceous nuclear matrix network, which later are exposed to high pH, to allow DNA to unwind. After this step, alkaline electrophoresis is carried out [9], attracting DNA nucleoids to the anode, but only DNA strands containing breaks migrate in the direction of the electrophoresis anode, generating comet-like shapes, giving the name to the assay [15,20]. The comet’s “head” contains undamaged DNA unlike the comet’s “tail” which contains damaged/relaxed DNA, which can be observed, usually, through fluorescence microscopy. Several authors argue that there is a direct proportion between the degree of intensity of the comet “tail” and the amount of DNA strand breaks existing in the individual cells [15].

The alkaline comet assay is mainly based on seven steps, namely, preparation of the microscope slides, lysis of cells, exposure to alkali (pH > 13), electrophoresis (pH > 13), neutralization of alkali, DNA staining and comet visualization, and finally the comet scoring.

### 2.1. Preparation of the Slides for Microscopy

The procedure begins with the preparation of the microscope slides, with the previously prepared suspension of cells to be analyzed. This step is performed to obtain the gels that should be enough stable to survive through the analysis, as well as to facilitate the visualization of the comets with a minimum background noise [19]. There are several techniques for the slides’ preparation, but they all involve embedding cells in agarose layers. Each slide can be prepared with one to three layers of one or two independent agarose gels [19].

The single-layer procedure consists in suspending cells in low melting-point agarose (generally 37 °C) and then placing them directly on the slide [19]. In the two-layers procedure, the slides are firstly pre-coated with a layer of regular agarose (these pre-coated slides are commercially available) and then an agarose layer containing the cells is placed on that pre-coated slide [19]. This first-layer coat of agarose on the slide improves the attachment of subsequent agarose layers. In the three-layers procedure, the process is very similar to the two-layers, but the difference is that the third layer with a Low Melting Point (LMP) agarose is added to increase the distance between the gel surface and the layer containing the cells, as well to ensure that any residual holes are removed of the second agarose layer [19].

There is a successful three-layers procedure that is known for generating stable gels and this success is mainly due to the concentration of the agarose gel as well the cells concentration [19]. The first layer, which is coating the microscope slide, usually has a concentration between 1% and 1.5% and it is dried at 40–50 °C [19]. The second layer, which contains the cells, is usually between 0.5% and 1% agarose concentrated, and it is added to the first a few days later. Finally, the third layer is at the same concentration as the second [19]. Usually, only a few cells are needed to perform the Comet Assay because a higher density of cells can result in comets overlapping, compromising the image analysis. The extent of DNA migration can also be influenced by higher agarose concentrations [19].

### 2.2. Lysis of Cells

This step consists of putting the agarose slides solidified in a lysis solution for the elimination of the membranes and to solubilize nuclear and cell constituents, forming the “nucleoids” (DNA attached to the nuclear matrix) [13]. Generally, the slides are in the solution (lysis buffer) for at least one hour, at 4 °C [13], but this period depends on the cell type. This lysis solution is composed of highly concentrated salts and detergents (e.g., EDTA, Sodium Chloride, DMSO, Triton X-100) and its composition also depends on the cell type. There are different types of lysis solutions according to different authors [19].

### 2.3. Alkali Unwinding

At the end of the lysis, the “nucleoids” comprising DNA at a highly condensed state [13] are incubated with an alkaline (pH > 13) electrophoresis buffer (EDTA and Sodium Hydroxide, pH > 13) [19] in order to produce single-stranded DNA and to express ALSs as SSBs. For most of the purposes, it is demonstrated that 20 min are enough for alkali unwinding, but this length of time varies between studies and among researchers [19].

### 2.4. Electrophoresis

After alkali unwinding, the next step is the electrophoresis under alkaline conditions, using the same pH buffer as the previous step. Usually, it is performed for a short period of time (20 to 30 min) [13] and conducted at the temperature of 5 °C to room temperature, depending on the cell type and the finality of the experiment, although the use of lower temperatures is thought to provide reproducibility increasing [19]. The typical voltages for electrophoresis are low, with the recommended voltage gradient ranging from about 0.5 to 1.47 V/cm [18].

### 2.5. Neutralization of Alkali

After electrophoresis, the neutralization step occurs, which consists in neutralizing the alkali in the gels with an appropriated buffer (e.g., PBS) [13]. Usually, three washes of the slides with the buffer are sufficient but if a high background is seen during scoring, additional rinsing may be beneficial [19]. After the neutralization, comets can be scored immediately or later, when convenient. However, slides should be scored with a reasonable length of time (e.g., 24 h) to prevent DNA excessive diffusion in the gel [19].

### 2.6. Staining of DNA and Visualization of the Comets

DNA staining is usually performed with fluorescent dyes, such as ethidium bromide (one of the most commonly used [15]) or 4′,6-diamidine-2′-phenyl indole dihydrochloride (DAPI), followed by visualization in fluorescence microscopy. However, this selection largely depends on the researcher’s specific needs and, depending on the dye (e.g., Ethidium bromide, SYBR Gold, SYBR Green I and II, SYBR Safe, Eva Green) [21], certain types of DNA strand breaks can be better visualized [15]. Table 1 shows different dyes and their use in visualizing the certain DNA strand breaks. Then, the fluorescence can be measured on a fluorescence microscope equipped with specific detectors or a digital camera [13]. Non-fluorescent techniques for comets visualization include staining DNA with silver nitrate [19], which demonstrated to increase the sensitivity/reproducibility of the assay when compared to the fluorescent staining [18]. Furthermore, it is also recommended to perform scanning of the gel so that the comets can be selected. This selection is very important because those comets will represent the whole gel, therefore, this procedure should be as narrow as possible [19]. The presence of comets around areas with air bubbles should be avoided, as well as comets with big tails and increased density of cells in the agarose (gels should have less than 2 × 10^4^ cells) [15]. Variability in the imaging and analysis of comet assay samples may result from variations encountered in the protocol implemented to process the cells, the system used form capture microscope images and the software for computerized analysis [22].

### 2.7. Comet Scoring

There are several different software packages and methods for quantifying the migration of the DNA by this assay. An image analysis technique for individual cells is a very suitable approach for comet scoring and analysis [13,18]. However, other systems are as useful [19], such as tail length, the relative fluorescence intensity of tail (normally expressed as % of DNA in the tail), and tail moment, whose parameters are not based on image analysis [15].

The most useful parameter applied is the relative fluorescence tail intensity as it gives a clear indication of what the comets actually looks like and it represents the intensity of the comet tail relative to the total intensity (head plus tail) [13]. Additionally, it allows discrimination of damage over the widest possible range, it is relatively unaffected by threshold settings and it reflects a linear correlation with break frequency [15]. The tail length is defined as the distance from the center of gravity of the nucleus, i.e., the position of the maximum fluorescence intensity over the nucleus, to the end of the tail. The tail moment is defined, essentially, as the product of DNA in the tail and the tail intensity [10]. However, these procedures are not as recommended as relative fluorescence tail intensity [15].

Another approach consists in evaluating comets’ appearance, directly through observation with the human eye (visual scoring), in five levels of damage, from zero (no tail) up to four (in which most of the DNA is present in the tail) gives enough resolution [15]. It is also a fast and simple method, which can be a suitable choice if the aim is to avoid expensive methods [19]. It was demonstrated that computer scores and visual scores have a high correlation between them [15].

## 3. Limitations of Comet Assay and Toxicological Assessment

There are still some factors that may create doubts about using comet assay to evaluate nanoparticles genotoxicity. Comet assay was firstly developed to detect DNA damage induced by soluble chemicals, and what happens is that nanoparticles are not removed, remaining during the assay [16]. Therefore, it is thought that nanoparticles can generate false levels of damage and that their presence within the nucleoid could affect DNA migration, as they are present in or in contact with cells, during the comet assay [16]. Ferraro et al. addressed this concern, by running the assay in the isolated nuclei instead of in the whole cells and concluded that this method resulted in an approximated result of the degree of genotoxicity induced by the nanoparticles, compared to the conventional one [16]. However, recent studies have shown that comet assays in vivo may even be superior to the well-established micronucleus erythrocyte assay as it can be applicable to any organ [24].

Nanotoxicology emerged as a multidisciplinary science [25,26,27], attributed to the urgency in evaluating the potentially harmful effects of nanoscale materials to biological systems, as well as the severity and frequency associated with the organisms and environment exposure to nanomaterials [28,29]. This need resulted from the fact that physical and chemical properties of nanoparticles are different from the respective bulk materials [30], together with the market growth of these materials [31].

There are several parameters that can affect the nanotoxicity profile of drug carriers but the most relevant ones are the size, shape and surface area, the surface characteristics, their stability, the impurities that compose the raw materials as well as their manufacturing methods, and the routes of exposure [32,33,34,35,36]. The size and surface area of nanocomposites are characteristics that have a huge impact on how they interact with cells because studies indicate that the higher the reduction of their size, the more toxic and reactive they become [37]. This happens because an increase in the superficial area/volume ratio occurs and consequently the risk of interacting with cellular organelles becomes bigger [37]. Therefore, nanoparticles with smaller dimensions have a higher capacity to, for example, reach the cells core and the increased possibility to interact with DNA, being more likely to cause DNA damage.

In terms of route of exposure, there are different barriers that a nanoparticle formulation needs to overcome, in order to achieve the target. Since the comet assay is a straightforward approach for nanoparticles genotoxicity testing in cells, its application in the nanotoxicology assessment field is becoming more frequent [38]. To demonstrate its continuous increasing practice, several studies on different types of nanoparticles are summarized in Table 2.

## 4. Evaluation of Genotoxicity of Metal Nanoparticles ccording to the Administration Route

### 4.1. Oral Administration

Oral nanoformulations for drug delivery are commonly used to protect drugs from proteolysis or to formulate poorly water-soluble drugs with the aim to increase their bioavailability through the gastrointestinal tract [43]. These nanoparticles can suffer systemic absorption and be captured by macrophages, that are present in many organs, e.g., liver, spleen, and kidneys, where nanoparticles can accumulate and cause toxicity [44]. Since the liver is the organ where the first-pass metabolism occurs, it is particularly vulnerable to the toxicity induced by nanoparticles, as these carriers can easily accumulate there, even long after risk of exposure [45]. On the other side, it has already been described that those nanoparticles can also be absorbed through the gastrointestinal tract via the lymph nodes, thereby undergoing transmigration to the liver and spleen [46,47,48]. The gastrointestinal tract can also be affected by the accumulation of nanoparticles [49]. Some experimental models commonly incorporated in the studying of the toxicity of ingested nanoparticles include intestinal epithelium cells (e.g., Caco-2, HT29, and SW480) [45].

Despite these characteristics, there is still limited information about their toxicological profile. Iglesias et al. evaluated the capacity of two types of poly(anhydride) nanocomposites, namely Gantrez^®^ AN 119-NP (GN-NPs) and Gantrez^®^ AN 119 covered with mannosamine (GN-MA-NPs), and their main bulk material (Gantrez^®^ AN 119-Polymer), to induce DNA damage in L5178Y TK^+/−^ mouse lymphoma cells, after 24 h of exposure to different concentrations [50]. In order to evaluate the possible genotoxicity of these nanoparticles and bulk material, the comet assay was performed in combination with formamidopyrimidine glycosylase (FPG), with the aim to check the presence of altered bases, DNA strand breaks (SBs) and alkali-labile sites (ALS) [50]. The 250 nm-sized particles, of negative surface charge and polydispersity index below 0.2 were not genotoxic to Caco-2 cells. Results showed that GN-NPs and GN-MA-NPs did not induce significant SBs nor ALS and FPG-sensitive sites in mouse lymphoma cells, which were shown to be more sensitive to nanoparticles than Caco-2 cells. On the other hand, the GN-Polymer was more effective in increasing the sensitivity to FPG, at the highest tested concentration (600 μg/mL) [50]. These findings allow the confirmation of the oral safety profile of the empty poly(anhydride) nanocomposites, by genotoxicity evaluation [50].

Magnesium oxide nanoparticles (MgO-NPs) are very attractive due to their unique properties, extensive applications and chemical stability. However, despite these characteristics, there is still limited knowledge about their safety profile and human health impact [51]. Mangalampalli et al. studied the in vivo acute toxicity of MgO-NPs and MgO microparticles (MgO-MPs) intended for oral delivery in female albino Wistar rats together with the genotoxicity assessment using the Comet assay [51]. Both types of particles presented an average size of 53 nm and 12 μm, respectively. The rats were treated with increasing dosages of these particles (100, 500, and 1000 mg/kg). The whole blood was withdrawn from the retro-orbital plexus of the animals, at various sampling times (24 h and 72 h), and liver tissues were isolated after sacrificing [51]. Peripheral blood lymphocytes (PBL) and liver cells were analyzed through the alkaline comet assay, showing that both of them presented a significant increase in % tail DNA at 1000 mg/kg dose of MgO-NPs, at the 24 h and 72 h sampling times. At the dose of 500 mg/kg, the MgO-NPs induced a significant % tail DNA at both sampling time-points in liver cells, whereas in PBL were only at the 24 h sampling time. When administering MgO-MPs, no significant damage was observed in all tested doses. Additionally, a gradual reduction of the % tail DNA was observed over time, attributed to the mechanisms involved in the complex DNA repair [51]. This study confirms that particles size is a very important characteristic from a toxicological standpoint, as it showed that nanoparticles induced higher genotoxicity than microparticles.

### 4.2. Skin Administration

The skin is one of the largest organs of the body and functions as a primary barrier between the external surroundings and the internal organs [45], becoming, therefore, an important route for contact with nanoparticles [52].

Nanoparticles are applied topically, they can potentially penetrate the skin, reach the blood circulation, and induce adverse side effects [45]. However, research has shown that nanoparticles typically do not penetrate into the dermal layers, which demonstrates that in intact skin it is unlikely for nanoparticles to penetrate the deeper layers of the skin. If the skin is however compromised with lesions on the surface, it is highly probable that nanoparticles can reach the blood circulation [53,54,55].

The toxicity of nanoparticles that enter the body through the dermal route is normally studied in fibroblasts, keratinocytes, and, more rarely, sebocytes (cells of sebaceous glands) [45].

Titanium dioxide nanoparticles (TiO_2_) are worldwide used in several areas, including as a coating material in pharmaceutical nanocomposites [52]. Furthermore, their properties make them very appealing as an ingredient for sunscreens and other cosmetic formulations, as these nanoparticles have UV-light blocking abilities, offer higher transparency, and better appearance to creams [45,52]. Amongst the potential exposure routes, nasal and skin exposure are considered the most relevant for NPs. Several studies clearly document that TiO_2_ nanoparticles can induce oxidative stress and DNA damage, as genotoxicity cellular effects [52,56].

Shukla et al. used human epidermal cells to evaluate the cytotoxicity, genotoxicity, and uptake of TiO_2_ [52]. The cells were exposed for 6 h to different concentrations of TiO_2_ nanoparticles suspension, to detect oxidative DNA damage in specific bases. The results showed that the DNA damage was enhanced at the three highest tested concentrations (0.8, 8, and 80 μg/mL) [52]. In commercial sunscreens, the concentration of TiO_2_ nanoparticles is commonly higher than the tested concentrations; however, considering that nanoparticles may remain onto the skin surface even after the formulation is cleaned up from the skin, the remaining particles can enter into this tissue and cause some damage. This study demonstrated that TiO_2_ nanoparticles may induce genotoxicity in human epidermal cells [52].

As happens with TiO_2_ nanoparticles, Zinc Oxide (ZnO) nanoparticles are widely employed in several industries, including cosmetics, personal care products and sunscreens, mainly due to their ultra-violet (UV) light absorption and antimicrobial properties [57,58]. ZnO nanoparticles constitute a type of metal oxide nanoparticles with promising applications in cell imaging, drug targeting and delivery. Their photocatalytic and photo-oxidizing properties against chemical and biological species, make these particles very appealing to figure in cosmetics, as food additives and in personal hygiene products. Additionally, zinc is proven to stimulate the immune system and demonstrated anti-inflammatory abilities. Recent studies showed that ZnO nanoparticles can induce cytotoxicity effects, followed by oxidative stress and genotoxicity, in leukemia and hepatocarcinoma cells in vitro, suggesting their application for the treatment of cancer therapy [59].

Studies demonstrate that ZnO nanoparticles are highly reactive compared to their bulk-sized materials, having those properties enhanced and may induce oxidative stress and genotoxicity in human cells. The fact that these nanoparticles are present in sunscreens (generally between 4 and 30 wt%) and exposed to UV radiation, along with the fact that they may induce ROS generation, encouraged Pal et al. to investigate the capacity of ZnO nanoparticles to induce DNA damage in primary mouse keratinocytes (PMKs), along with UVB-exposure [57]. ZnO nanoparticles of 32 nm of mean size and zeta potential of −9.21 mV, were in contact with PMKs for 24 h, at the concentration of 1 μg/mL. The comet assay was carried out in PMKs exposed to UVB alone, to ZnO nanoparticles alone, and to a combination of both. Results showed that tail moments value was greater in the combination groups compared to ZnO nanoparticles and UVB alone [57]. Sharma et al. evaluated the genotoxicity of ZnO nanoparticles of 30 nm of mean size and −15.8 mV of surface electrical (zeta potential) on the most abundant cell type in human epidermis i.e., primary human epidermal keratinocyte. At the tested concentration of 14 μg/mL ZnO nanoparticles induced significant genotoxicity on those cells, when in contact for 6 h [60].

Quantum dots (QDs) are fluorescent semiconductor crystals composed of a semiconductor inorganic core, an inorganic shell, and an aqueous organic coating. This latter improves their water solubility, stability and bioactivity [61,62]. Their diameter is usually between 1 and 10 nm, and the core is composed of metal elements from the groups II–V, with Cadmium as one of the most commonly used elements [61,62,63].

Several studies demonstrate that Cd is highly toxic and with the capacity to induce ROS formation, DNA damage and cell death [64,65].

To study the genotoxic risk of QDs, Ju et al. used the neutral comet assay on QDs of two different sizes, 4–5 nm (QDs with a core/shell of CdSe/ZnS) and 8–10 nm (QDs with a core/shell of CdSe/ZnS coated with a PEG thin-layer). The study aimed to check the effect of the PEG coating on the induction of DNA damage compared to the non-coated QDs. The sizes were also according to the commonly available size range. Other studies have shown that nanotoxicity is dependent on the surface properties of nanocarriers; PEG-coating on QDs could be an approach to decrease their toxicity [61]. The uncoated QDs and PEG-QDs of two distinct sizes were applied at the concentrations of 8 nM and 80 nM, in human skin fibroblasts for 8 h. After 2 h of exposure, uncoated QDs induced significant DSBs at both concentrations, with an increase in the tail moment at the highest concentration, while PEG-QDs showed no significant changes in results of the tail moment, compared to the control [61]. These outcomes encourage the fact that a proper surface modification in QDs can make a difference in their interaction with skin cells. In fact, the PEG-coating layer may prevent cadmium leakage, thereby reducing the generation of ROS by QDs and therefore reducing possible genotoxicity induction. Moreover, the uncoated QDs induce genotoxicity in a dose- and time-dependent manner. The long-term exposure to QDs still requires further investigation [61].

### 4.3. Pulmonary Administration

The first-pass metabolism can avoid the systemic side effects when using the pulmonary route for systemic drug delivery [45]. However, because of their large surface area, nanoparticles may enhance the risk of inducing toxicity over non-loaded drugs, as the particles can accumulate in the lung tissue to a large extent. Studies have shown that nanoparticles with the size of about 50 nm can lead to membrane perforation of type 1 alveolar cells, resulting in nanoparticles internalization in these cells [45]. The toxicity of inhaled nanoparticles is commonly studied using model cell lines that differ from respiratory system tissues, e.g., A459 and C10 cells of pulmonary origin, alveolar macrophages, various epithelial cells and fibroblasts and also human monocytes, posing an additional problem on the assessment of the cyto-genotoxicity [45].

The International Agency for Research on Cancer has listed carbon black particles among the substances with the potential risk of carcinogenesis in humans and yet carbon black is applied largely in the chemical industry for the production of rubbers, toners, paints. There is therefore a risk of occupational exposure through inhalation of these particles during the handling of dry powders [66]. Studies have shown that carbon black nanoparticles have the capacity to induce ROS and cause DNA strand breaks in the lungs.

Kyjovska et al. studied this possibility by administering, by intratracheal instillation, a single dose of 0.67, 2, 6, and 162 μg/animal of carbon black nanoparticles (size: 14 nm) to mice (8 mice for each dose). The animals were killed 1, 3 or 28 days after exposure to nanoparticles, and their lungs, liver and Broncho-Alveolar Lavage (BAL) were collected to run the alkaline comet assay [66]. The results demonstrate that there was DNA damage in the BAL, after one day of exposure to 0.67 and 2 μg/animal and it was significant for the 0.67, 2, and 6 μg/animal dose, 28 days post-exposure [66]. In the lungs, there was no significant DNA damage on the three lower concentration groups, after one day of exposure, but significant strand breaks were detected at the highest dose. After 28 days, 2 and 6 μg/animal caused a significant increase in the level of DNA damage [66]. In the liver, no DNA damage was detected at any doses and time-points of exposure. A lack of dose-response relationship was reported in this study [66].

Gold nanoparticles (AuNPs) have been attracting scientific interest not only for the facilities in their synthesis and surface bioconjugation but also for their unusual optical, electronic, and thermal properties [67]. These nanoparticles have large medical applications. However, their possible nanotoxicity effects are still unknown [68]. Ng et al. studied the risk of AuNPs of 20 nm in inducing genotoxicity on small airway epithelial cells (SAECs), exposing these cells to concentrations of 1 nmol/L (equivalent to 48.65 μg/mL) to AuNPs, through the in vitro alkaline comet assay, for 72 h [68]. DNA damage was observed at this concentration and demonstrated when compared to the control, a significant increase of the tail moment [68].

### 4.4. Ocular Administration

One of the major challenges for drug delivery has been ocular administration, particularly when it comes to nanoformulations, due to intricate and unique anatomical and physiological barriers in the eye, that protect it from the invasions of microorganisms and environmental toxicants, keeping the systemic circulation from the ocular tissues [69,70,71,72].

These barriers make the eye a highly protected organ and therefore when an ocular disease occurs, it becomes very difficult to set a treatment, especially in the ocular posterior segment [70,71,73]. To treat this area, several delivery modalities have been applied, such as intravitreal injection, the most commonly applied method for posterior drug delivery. Subretinal injection, subconjunctival injection and topical administration are also used. However, these are not satisfactory since they are invasive procedures with serious associated risks. Therefore, a better approach is still required [69,74].

Current developments in nanoparticles drug delivery have become promising traits for the prolongation of the drug release and to enhance drug retention/permeation in ocular tissue, providing novel opportunities to overcome the limitations of conventional drug delivery systems [69].

Particles larger than 1 μm may potentially cause ocular irritation [70,71]. Therefore, nanoparticles for ocular installation may be an advantage to reduce the irritation of the eye, as well as to enhance the bioavailability of topical administration, achieve controlled release, targeted delivery, reduce the frequency of administration with improved patient compliance, and ultimately, improved therapeutics efficacy [69].

To explore the use of ZnO nanoparticles in ocular drug delivery and the risk of inducing genotoxicity in ocular tissue, Guo et al. performed a study, using a RGC-5 cell line, since the rat retinal ganglion cells were more susceptible to outer surroundings than other eye cells [59]. The cells were exposed to different concentrations of ZnO nanoparticles (0, 2.5, 5.0, and 10.0 μg/mL) of a mean size of 100 nm for a 6 h period. The results show that untreated cells had an intact nucleus, with no formed comets unlike the treated cells, for which the damage was increased with the increase of the concentration of ZnO nanoparticles [59].

Cerium oxide (CeO_2_) nanoparticles are known for their antioxidant and optical properties and for having a high affinity to oxygen. These nanoparticles may constitute a potential means for imaging and drug delivery to the ocular tissue for the treatment of e.g., cataract and glaucoma. Studies involving the effects of CeO_2_ nanoparticles on eye lens suggest that these particles may have a protective effect on the retina [75]. The outcomes of these nanoparticles in vivo are however not well known, since their application in medicine is a new field to be exploited. It is therefore mandatory to further characterize the possible toxicological effects of these particles in the eye (e.g., potential risk of DNA damage), as they can influence the formation of structural proteins and eye cells negatively, leading to potential diseases [75].

Pierscionek et al. use the alkaline comet assay in three replicated cultured human lens epithelial cells, incubating the cells with two sets of CeO_2_ nanoparticles of mean size of 5.5 nm, i.e., one set with the concentration of 5 μg/mL and the other set with the concentration of 10 μg/mL [75]. The comets were scored by % of DNA tail and head, tail length, and olive tail moment and results demonstrated a low level of DNA damage in all data sets. When applying the highest dose, there was a slight increase in the % of DNA tail, however, no statistical differences were recorded between control and treated cells for both tested concentrations [75].

### 4.5. Parenteral Administration

Parenteral nanoparticles are applied as therapeutics and diagnostics, as drug carriers and contrast agents, respectively. Nano-intravenous administration is a very significant route used in defining toxicological profiles of nanoparticles, in biological assessment. Several studies established that there is a high probability of occurring deposition of nanoparticles in several organs through this type of exposure [76]. The toxicity of these particles is usually studied in primary blood cell cultures, mononuclear blood cells, cultured HUVECs, mesenchymal stem cells, and various tumor cell lines (HeLa, MCF-7, PC3, C4-2, and SKBR-3) [45].

Silver nanoparticles (AgNPs) are amongst the most commercially used nanocomposites. These metallic nanoparticles have attracted technical interest and intense scientific due to the optical, electronic, and thermal properties that make these nanoparticles unique. The easy surface bioconjugation and synthesis make these systems very pleasant for drug delivery. They are well-known not only for being excellent antibacterial and antiviral agents but also for having a great performance as anti-angiogenic agents, with applications in multiple myelomas, leukemia, and rheumatoid arthritis [77].

With the increasing use of AgNPs, their safety and potential risk to human health have been discussed and raised, therefore, scientific research is required to evaluate the potential toxicity and the genotoxicity of these nanoparticles [77].

Several in vivo studies, although in a limited quantity compared to the in vitro ones, have been carried out, in order to evaluate genotoxicity of AgNPs in the body tissues [77], as in vitro data alone may not be sufficient for genotoxicity assessment of nanocomposites [77]. It is reported that AgNPs might generate reactive oxygen species (ROS) when accumulated in the liver, causing hepatotoxicity [78].

Li et al. tested two in vivo types of silver nanoparticles (AgNPs), namely PVP-coated AgNPs (of 5 nm mean size) and silicon-coated AgNPs (of 10–80 nm mean size). Particles were administered intravenously to 7-week-old male mice (weighing 25–30 g). for three consecutive days to evaluate the possible effect of size and coating. Additional groups of mice served as negative and positive controls [77]. For both PVP- and Silicon-coated AgNPs, no DNA strand-breaks were detected in the liver when using the standard Comet Assay, while a significant induction of DNA damage was found in the enzyme-modified Comet Assay, with silicon being the most toxic to the cells. The addition of nuclease enzymes resulted in DNA breaks which suggest that AgNPs can cause oxidative DNA damage [77].

The genotoxicity of AgNPs in the liver of rabbits was tested by Kim et al. one week and one month after a single intravenous injection into the ear veins. The tested nanoparticles had a citrate coating (cAgNPs) which offers a negative charge on the particles’ surface. The size of cAgNPs was approximately 7.9 nm [78]. The suspensions of cAgNPs comprehended a low dose of 0.5 mg/kg and a high dose of 5 mg/kg that were given, respectively, to two groups of four rabbits. The results demonstrated that the damage of DNA in liver tissue was higher in the group of the 5 mg/kg dose than in the 0.5 mg/kg. Plus, the DNA damage at day 28 declined compared to the damage at day 7, in the high-dose treated group, which reveals time- and dose-dependent variations in genotoxicity and oxidative stress, after a single injection of the tested particles [78].

TiO_2_ nanoparticles, as discussed above, are largely used in industry, and because of their multiple applications, it becomes necessary to investigate every possible form of these nanoparticles interacting with the human body and the possibility of inducing genotoxicity [79], as humans are being increasingly exposed by multiple routes [80]. The risk of these particles reaching the endothelium is almost inevitable and can occur before reaching other secondary organs, which can cause endothelial dysfunction and impairments, consequently affecting cardiovascular health [79,80].

Liao et al. investigated the effect of TiO_2_ nanoparticles on the cardiovascular system, evaluating the genotoxic potential of four sizes (100, 50, 30, and 10 nm) of TiO_2_ nanoparticles in HUVECs, through the comet assay and exposing the nanoparticles to the cells through 4 h [79]. All the sizes demonstrated to induce DNA damage which decreased with the size increase of TiO_2_ nanoparticles, revealing the importance of studying the size effect on inducing cellular responses [79].

Iron oxide nanoparticles (IONPs) are being used in biomedicine due to their magnetic properties, possible use as carriers for gene delivery, and in cancer therapy. Ansari et al. performed the comet assay in vivo on male Wistar rats with the administration of IONPs through the intraperitoneal route, for 7 consecutive days. The animals were split into 9 groups, each one with six animals. Three groups were studied with three different IONPs concentrations (25, 50, and 100 mg/kg) respectively [81]. IONPs were characterized for their size and shape, showing that they had a spherical shape and an average size of approximately 60 nm [81]. In order to perform the comet assay on the lymphocytes of the rats, the animals were sacrificed, and their blood was freshly collected to isolate the cells for the assay [81]. Results showed that the average tail length increased with the increase of the IONPs concentration compared with the negative control, with the concentration of 100 mg/kg having the higher average tail length.

## 5. Conclusions

Over the years, the diversity and the complexity of nanocomposites have been growing, however, the respective translation into the clinic has been limited. The possible causes for this limitation may be the lack of established characterization and testing regimes that can provide regulatory authorities (e.g., FDA, EMA) with the necessary data to allow novel nanocomposites reach the market. There are currently no tangible strict guidelines regarding toxicity testing for nanoparticles and, therefore, the implementation of new characterization technologies or the adaptation of currently available ones should be done in close interaction with regulatory authorities, to ensure that the new assimilated data on candidates will therefore allow novel nanomedicine to be part of healthcare advances and create a difference on a global scale. In general, the strategies used to evaluate the safety/toxicity and biocompatibility of nanocomposites have been adapted from the techniques applied in the testing of conventional drug products and, therefore, there is an urgency in proving that these methods are adaptable and viable for nanotoxicity evaluation. The same characteristics that make nanocomposites interesting for many applications are the same that lead to genotoxicity effects. The assessment of genotoxicity of nanoparticles can benefit from the comet assay, not only because of the characteristics of the assay but also because the current standardized practices used for assessing the genotoxicity of chemicals not always are proper for nanogenotoxicity assessment. According to OECD, an assay that identifies and characterizes DNA damage is required not only through direct interaction, detecting DNA strand breaks and altered DNA bases but also through indirect and secondary mechanisms as well (e.g., oxidative stress induced by inflammation). Comet assays meet these requirements and may therefore be a suitable approach to include in upcoming guidelines for nanotoxicity assessment.

## Figures and Tables

**Table 1:** Brief characterization of several dyes used for the visualization of the DNA strand breaks (modified after [23]).

Name of Dye	Characterization	Uses for Staining DNA
Ethidium bromide	− the most well-known dye used for visualizing DNA − it can be used in the gel mixture, the electrophoresis buffer, or to stain the gel after it is run	− molecules of the dye adhere to DNA strands and fluoresce under UV light, showing where the bands are within the gel
SYBR Gold	− more sensitive than ethidium bromide − used to stain double or single-stranded DNA − used to stain RNA	− the dye binds to nucleic acids showing high UV fluorescence
SYBR Green I	− may be mutagenic because they bind to DNA − more sensitive for double-stranded DNA	− requires careful handling − it fluoresces under UV light
SYBR Green II	− may be mutagenic because they bind to DNA − more sensitive for single-stranded DNA or RNA	− requires careful handling − it fluoresces under UV light
SYBR Safe	− it is less toxic on several human cells that other dyes	− it can be used with a blue light which causes less DNA damage
Eva Green	− suitable for low-melting-point gels − it shows very low or no cytotoxicity − it shows no mutagenicity	− it shows low fluorescence alone but high fluorescence when bound to DNA

**Table 2:** Examples of metal nanoparticles assessed by Comet assay for their risk of genotoxicity.

Type of Nanoparticles	Properties	Genotoxic Outcomes	References
Cerium dioxide nanoparticles (CeO_2_ NPs)	0.5, 2 and 10 μg/mL CeO_2_ NPs of 175.3 ± 10.2 nm tested in salivary leucocytes	Increased primary and oxidative damage; no changes in DNA migration during electrophoresis, either by inducing additional breaks into the naked DNA or inhibiting DNA migration	[39]
Gold nanoparticles (AuNPs)	30, 50 and 90 nm (1–10 μg/mL) AuNPs tested in tumoral human leukaemia cells (HL-60) and human hepatoma cells (HepG2)	In both cell lines, pyrimidines and purines were oxidatively damaged by all AuNPs, being 90 nm AuNPs slightly more genotoxic, using a endonuclease III and formamidopyrimidine-DNA glycosylase restriction enzymes modified comet assay	[40]
	Carboxylate-, ammonium- or poly(ethylene glycol-functionalized Au NPs cores of ~5 nm and ~20 nm mean size tested in human bronchial epithelial BEAS-2B cells	Cationic ammonium AuNPs were more cytotoxic than their anionic (carboxylate) and neutral (PEG)-functionalized AuNPs; 20-nm ammonium and PEGylated AuNPs induced DNA damage, while micronucleus induction was increased by 5-nm ammonium and 20-nm PEGylated AuNPs	[41]
Titanium dioxide nanoparticles (TiO_2_-NPs)	10 nm NPs at 200 µg/mL tested in TK6 cells	TiO_2_-NPs were taken up by TK6 cells without significant induction of DNA breakage or oxidative DNA damage using the standard alkaline Comet assay and the endonuclease III (*Endo*III) and human 8-hydroxyguanine DNA-glycosylase (hOGG1)-modified Comet assay	[42]
	80, 120 and 150 μg/mL TiO_2_ NPs of 199.1 ± 2.6 nm tested in salivary leucocytes	Increased primary and oxidative damage; no changes in DNA migration during electrophoresis, either by inducing additional breaks into the naked DNA or inhibiting DNA migration	[39]
Zinc oxide nanoparticles (ZnO NPs)	20, 30 and 40 μg/mL ZnO NPs of 485.6 ± 26.3 nm tested in salivary leucocytes	Increased primary and oxidative damage; no changes in DNA migration during electrophoresis, either by inducing additional breaks into the naked DNA or inhibiting DNA migration	[39]

## Data Availability

Not applicable.
